# Evaluating the Crossmatch-to-Transfusion Ratio as a Tool for Analyzing and Optimizing Blood Bank Resource Utilization: A Retrospective Observational Study

**DOI:** 10.7759/cureus.69862

**Published:** 2024-09-21

**Authors:** Vijit Joon, Rowena D.L. Robins, Hari Haran A, Suresh Kumar I, Sahayaraj James

**Affiliations:** 1 Transfusion Medicine, Saveetha Medical College and Hospital, Saveetha Institute of Medical and Technical Sciences, Chennai, IND

**Keywords:** blood products, cross-match to transfusion ratio, hospital transfusion committee, maximum surgical blood ordering schedule, packed red blood cells, patient blood management, transfusion

## Abstract

Background

Blood transfusion services are crucial for modern medical care, particularly for surgical interventions, chronic diseases, pregnancy complications, and malignancies. There is an increasing demand for blood products in India; hence, optimizing resource utilization in blood banks is essential. This study aims to evaluate the crossmatch-to-transfusion (C/T) ratio as a tool for analyzing and optimizing the utilization of blood bank resources. The C/T ratio of the various clinical departments will also identify inefficiencies in blood ordering practice and plan corrective actions.

Methods

A retrospective observational study was conducted at the Blood Center of Saveetha Medical College and Research Center. Data were collected over a three-month period from November 1, 2023, to January 31, 2024. Information was collected from crossmatch request forms, crossmatch registers, issue registers, and digital medical records. The study included all crossmatch requests received within the study period. The C/T ratio was calculated department-wise, and the departments with high C/T ratios were identified.

Results

In the study, 1,861 packed red blood cell (RBC) units were crossmatched, of which 797 units were issued. The overall hospital C/T ratio was 2.33, which indicates excessive blood ordering. The Department of Obstetrics and Gynaecology had the highest C/T ratio of 5.14, followed by Oncology (3.09), Urology (2.9), General Surgery (2.26), and Orthopaedics (2.08). These high ratios showed significant overordering and underutilization of crossmatched blood units.

Conclusion

This study revealed that the C/T ratio in the hospital exceeded international standards, especially among surgical departments. This indicates a need for optimizing blood ordering practices to reduce unnecessary crossmatches, minimize wastage, and enhance resource management. Implementing strategies to correct the high C/T ratio and regular auditing can improve blood transfusion services and ensure efficient utilization of blood products.

## Introduction

The first blood transfusion was recorded on December 22, 1818, by a physician/obstetrician named James Bundell [1]. At present, blood transfusion is an invaluable therapeutic intervention within a hospital. Blood transfusion services collect whole blood from healthy donors, prepare proper blood products with processing, and adequately screen the various components [2]. Transfusion services are essential in hospitals, treating patients for surgical interventions, chronic diseases, complications related to pregnancies, and malignant conditions. It is an important treatment modality in the resuscitation and management of the conditions mentioned above [3]. Thus, the services provided by a blood center are primarily therapeutic.

With each passing year, there is a progressive increment in the requirement for blood products in India [1]. This can be observed with the increased collection of whole blood from donors and the number of utilizations of the processed blood products by the patients. In 2019-2020, India collected nearly 12.5 million units of blood, but the demand was found to be 13.2 million units [4]. Blood and other components must be collected from healthy individuals, preferably from regular whole blood donors [3]. The blood donation rates are not enough to meet the demands of the patients within our country. Hence, the needs of the patients are not fully met. To counter this, the unnecessary blood crossmatches that affect the blood stocks should ideally be prevented [1].

It has been observed that there is a tendency for "over-ordering" blood components by clinicians, which ultimately affects the stocks in a blood center. These crossmatched blood units will be unavailable for patients requiring the blood product on a more urgent basis or for genuine indications. This is an issue of concern, which will negatively affect the already limited blood stocks within a blood center and the loss of the shelf life of the crossmatched blood products, thereby resulting in the wastage of precious resources [2]. Over-ordering blood products also leads to an increased workload burden for the blood center staff and an unnecessary financial strain on the patients. This practice will result in the exhaustion of valuable laboratory reagent supplies, resources, time, and staff manpower [1]. Therefore, it is preferable to critically and methodically assess the patterns of blood transfusion orders and to identify departments with a high crossmatch-to-transfusion (C/T) ratio [5].

The tendency of "over-ordering" is mostly seen among patients posted for elective surgery. These preoperative requisitions of the blood units are mostly based on the assumption of a worst-case scenario or overestimation of intra-op or post-op blood loss, which leads to unnecessary demands for a large number of units of packed red blood cells (RBCs) [1]. This practice of reserving and blocking a ration of packed RBCs by crossmatching in such cases will often lead to ineffective blood usage and may result in wastage of the reserved packed RBCs [6,7].

The C/T ratio is considered an essential quality indicator that can be used to measure the appropriateness or efficiency of blood ordering practices within the blood transfusion services in a hospital [1] and was first used by Boral Henry in 1975 [8], after which many authors have used the C/T ratio to analyze blood transfusion practices. The C/T ratio is defined as the number of packed RBC units crossmatched divided by the number of packed RBC units transfused. A value above 2 indicates excessive ordering, per the Association for the Advancement of Blood and Biotherapies (AABB). High C/T ratios will help identify the department's or clinician-specific blood-ordering patterns and determine whether crossmatching alternatives may be required [7]. The aims and objectives of this study were to assess the blood utilization practices using the C/T ratio, identify the departments with a high C/T ratio, and plan preventive and corrective actions for the departments with a high C/T ratio.

The AABB requires blood transfusion services to have a system that ensures appropriate usage of the valuable and scarce resources of the blood center [7]. A proper review and auditing of the blood ordering and utilization practices should be done by the Hospital Transfusion Committee (HTC). The collected data can be used for discussions at the HTC meetings, which should include all the concerned clinical and administrative departments [7], thereby improving blood transfusion services and implementing measures that can enhance the services provided by the blood center.

## Materials and methods

Study design

This study was a cross-sectional study at our blood center.

Setting

The study period spanned three months, from November 1, 2023, to January 31, 2024, and was conducted at the Department of Transfusion Medicine and Immunohematology of Saveetha Medical College and Research Center. This institution is a tertiary care hospital equipped with a 1160-bed facility, serving patients from various socioeconomic categories. The required data were collected from the packed RBC crossmatch request forms, crossmatch registers, issue registers, and digital medical records.

Participants

All blood crossmatch requests received during the study period and as per the eligibility criteria were included in the study. Our hospital's request forms included various information such as patient personal details, patient identification number, ward, the clinician treating the patient and the department, diagnosis, indication for transfusion, pre-transfusion hematological results, the required unit and the number of units, details regarding past transfusion, and pregnancies in the case of women. The patient's personal data were not recorded for this study.

Inclusion and exclusion criteria

The study included all crossmatch requests submitted to the blood center of our hospital from November 1, 2023, to January 31, 2024. Blood requests for hospital transfusions that were not within our hospital were excluded from the study.

The data collected were examined according to the total quantity of packed RBC requested, the utilization trends of packed RBC across different specialties, the therapeutic justification for each request, and the units that were crossmatched and transfused to patients. The collected data were categorized into the following departments: Cardiology, Nephrology, ENT, General Medicine, General Surgery, Urology, Oncology, Paediatrics, Vascular Surgery, Plastic Surgery, Neurosurgery, Orthopaedics, and Gynaecology and obstetrics.

Statistical methods

The packed RBC crossmatches were gathered on a department-wise basis, along with the corresponding usage of packed RBCs. Subsequently, the C/T ratios were computed for both the entire hospital and each individual department. The C/T ratio throughout the study period was determined using the following equation: C/T ratio = number of units crossmatched/number of units transfused. A ratio above two is considered excessive blood ordering. The department-wise calculated values were presented in tables.

## Results

Participants

**Figure 1:**
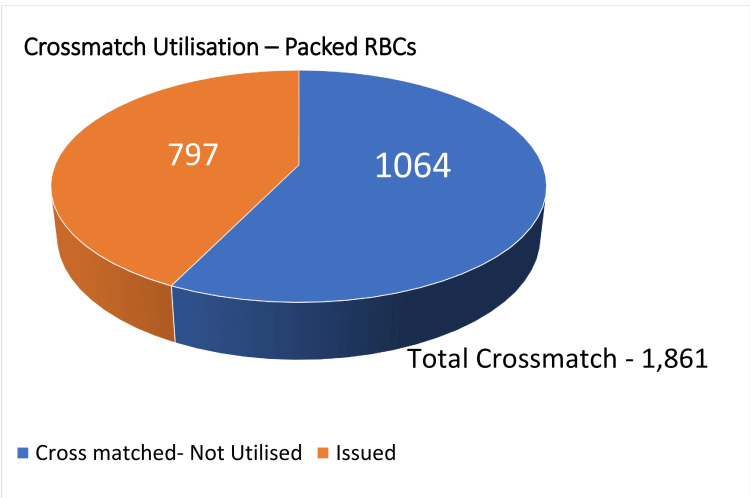
Number of crossmatch versus utilization of packed RBCs RBC: red blood cell

During the three-month study period, there were 1,861 packed RBC crossmatches among the various departments per the eligibility criteria. Out of the above crossmatched packed RBCs, 797 units were issued to the patients. These data are plotted in Figure 1.

Outcome data

**Figure 2:**
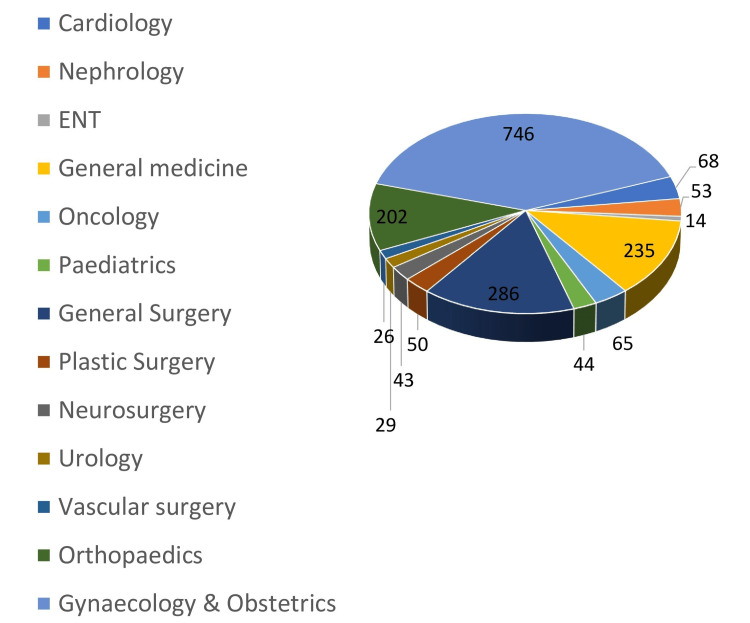
Department-wise number of crossmatches of packed RBC for patients ENT: ear, nose, throat; RBC: red blood cell

**Figure 3:**
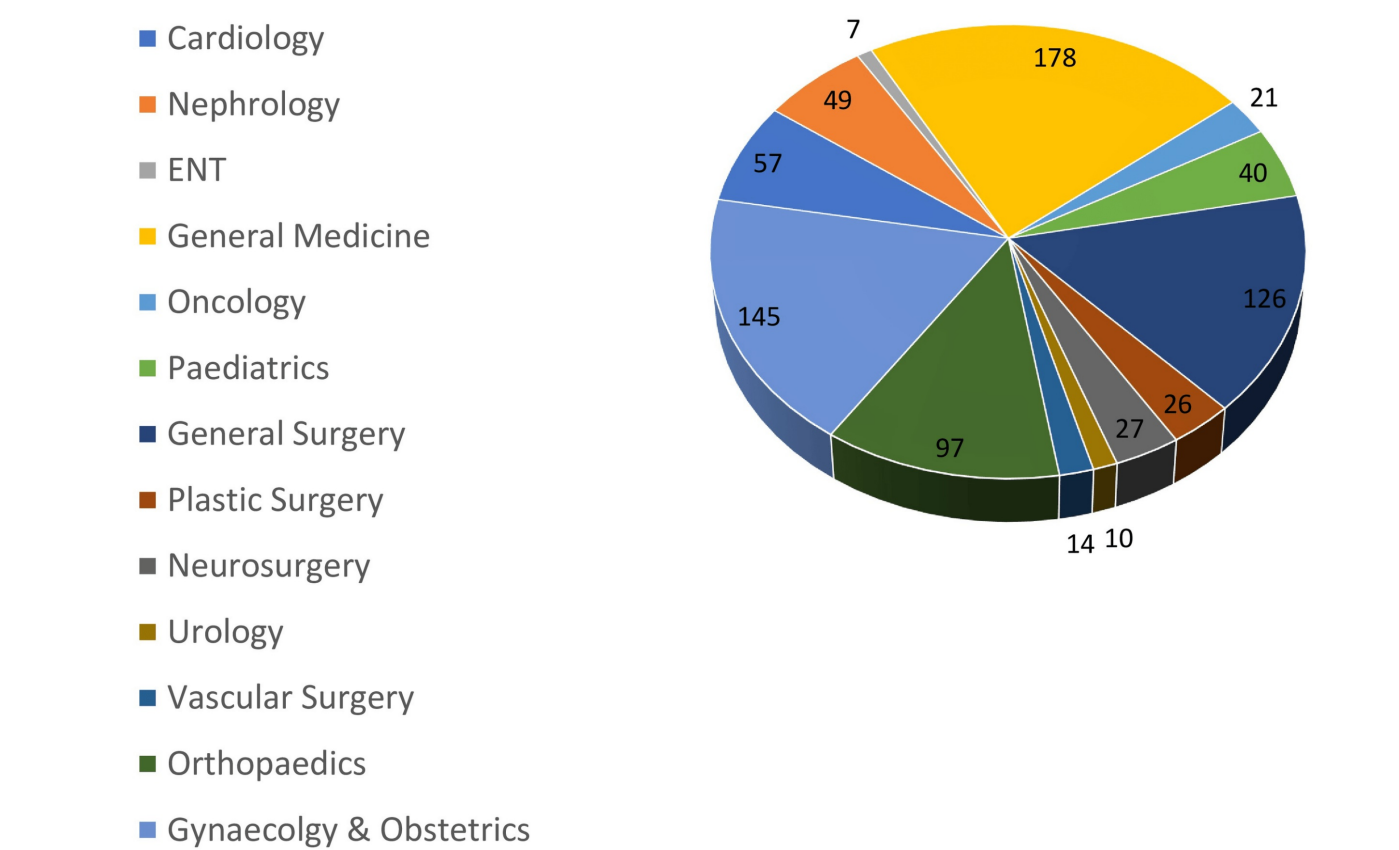
Department-wise number of utilization of packed RBCs ENT: ear, nose, throat; RBC: red blood cell

The department-wise total crossmatches are shown in Figure 2, and the department-wise total packed RBC utilization is shown in Figure 3. The Obstetrics and Gynaecology department had the highest packed RBC crossmatch requests of 746, of which only 145 were issued to the patients. The Department of General Surgery had the second-highest number of patients crossmatched, totaling 286 patients, of which 126 packed RBCs were issued. This was followed by the General Medicine department, which had crossmatched 235 patients, of which 178 units were issued to the patients.

**Figure 4:**
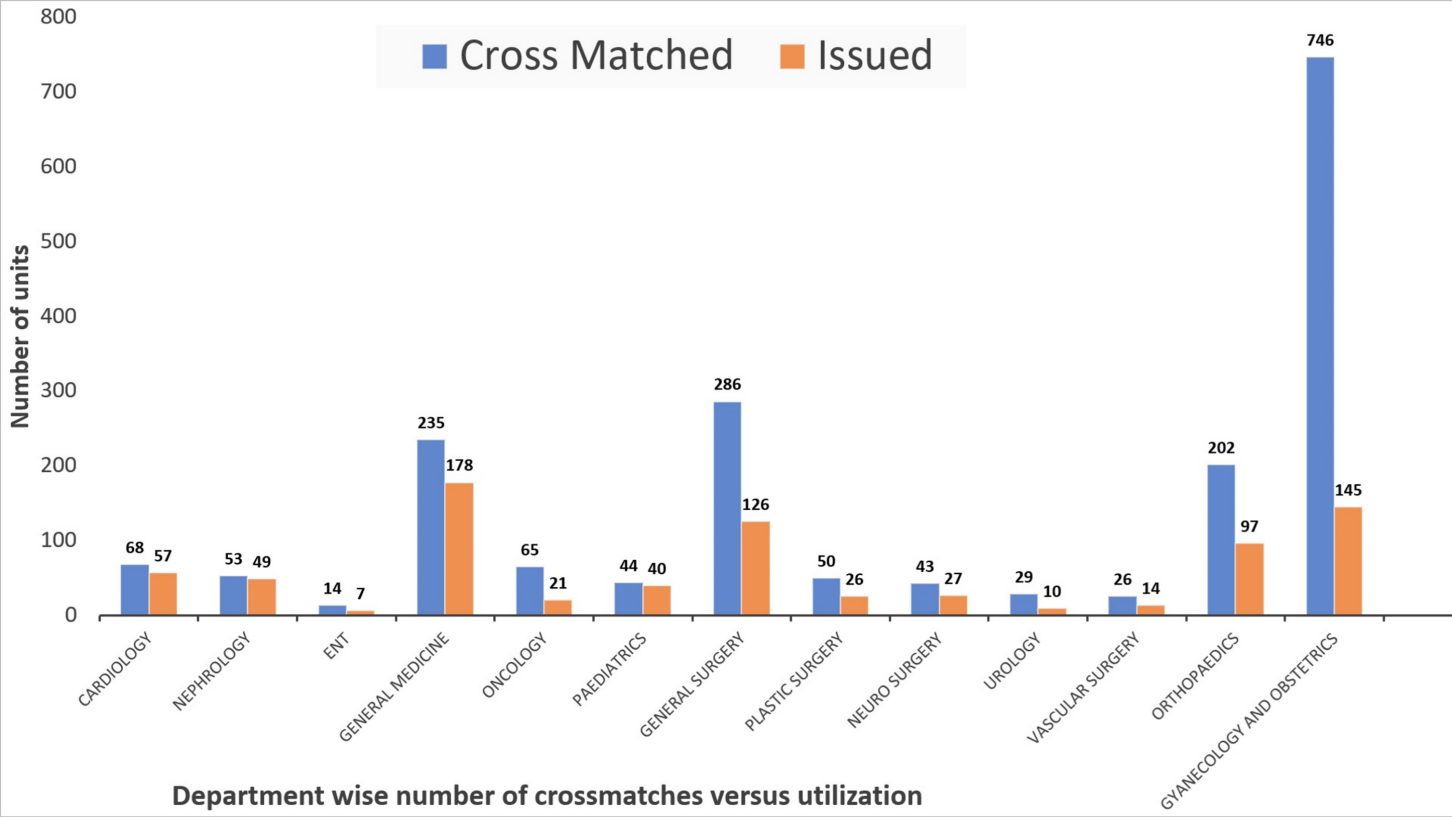
Department-wise number of crossmatches versus utilization ENT: ear, nose, throat

Figure 4 shows the department-wise crossmatching and utilization of the packed RBCs. This graph represents the distribution of crossmatch and utilization of packed RBCs.

The C/T ratio for each department was computed and is presented in Table 1. The calculated C/T ratio for the entire hospital was found to be 2.33, exceeding the value stipulated by the AABB. The highest C/T ratio was observed in the Obstetrics and Gynaecology department, reaching 5.14. This was followed by Oncology (3.09), Urology (3.09), General Surgery (2.26), and Orthopaedics (2.08).

**Table 1:** Department-wise C/T ratios C/T: crossmatch-to-transfusion ratio

Sl. No.	Department	No. of Crossmatches	No. of Transfused Units	C/T Ratio
1.	Nephrology	53	49	1.08
2.	Cardiology	68	57	1.19
3.	Paediatric	44	40	1.1
4.	General medicine	235	178	1.32
5.	Neurosurgery	43	27	1.59
­­6.	Vascular surgery	26	14	1.85
7.	Plastic surgery	50	26	1.92
8.	ENT	14	7	2
9.	Orthopaedics	202	97	2.08
10.	General surgery	286	126	2.26
11.	Urology	29	10	2.9
12.	Oncology	65	21	3.09
13.	Gynaecology and obstetrics	746	145	5.14
Total	-	1861	797	-

## Discussion

In our blood center, the crossmatch procedure for packed RBCs is a major crossmatch test, along with blood typing and screening of the respective patient's serum. Screening is done to detect any unexpected allo-antibodies in the serum of the patients, which is usually done when the samples arrive at the blood center for crossmatch the first time. The major crossmatch is testing for incompatibility of the patient's serum with donor cells, done with the gel card method. The typing procedure is done on the patient's cells, on which these cells are tested for ABO and Rd(D) with the respective anti-serums. The screening procedure is done with the patient's serum, which is tested for unexpected antibodies using single-cell or three-cell lines, usually done with the gel card method (the tube method may also be done). If any antibodies are detected, the particular antibody in the patient's serum will be identified.

The crossmatch procedure, on average, takes an hour, while an emergency crossmatch (if indicated) can be done within 30 minutes. Once a unit is crossmatched, it will be reserved for 48 hours, and that crossmatched unit will not be shown in the blood center's stock. This study was done to evaluate the effective utilization of blood transfusion services. For this, the C/T ratio was used.

Our analysis found that our hospital's overall C/T ratio was 2.33. Our institution's C/T ratio is above 2, which indicates excessive crossmatching of packed RBCs [7]. The C/T ratio value of 2.33 means that the blood center is overburdened by crossmatching approximately 2.33 times more blood than the blood being utilized.

The analysis of the department-wise C/T ratio revealed that the Obstetrics and Gynaecology department exhibited the highest value of 5.14, followed by Oncology with 3.09 and Urology with 2.9. General Surgery and Orthopaedics had C/T ratios of 2.26 and 2.08, respectively. The next highest C/T ratio was observed under the departments of Oncology (surgical and medical), Urology, General Surgery, and Orthopaedics, with values coming in 3.09, 2.9, 2.26, and 2.08, respectively.

When we analyze the result of the Obstetrics and Gynaecology department, the C/T ratio of 5.14 means that the blood center is overburdened by 5.14 times more crossmatch procedures than the blood being utilized. It can be observed that within the study period, the Department of Obstetrics and Gynaecology had a total of 746 crossmatches sent to our blood center, of which only 145 were issued to the patients. The total crossmatches comprised up to 40% of the total crossmatches within the hospital during the study period, while the utilization was only 18.33% of the packed RBCs utilized within the study period.

Similar results were seen in a study by Kaur et al. [10], but the highest C/T ratio in that study was only 3.6:1 in the Obstetrics and Gynaecology department. However, a study done by Trisal et al. in 2020 had the highest C/T ratio of 2.7:1 from the department of ENT [13]. Another similar study by Kumari et al. had the highest C/T ratio of 2.8:1 in the Department of Gynaecology and Obstetrics [12]. The analysis reveals that the C/T ratio at our institute is elevated, suggesting a tendency towards over-ordering practices.

Upon analysis of all departments exhibiting a high C/T ratio, it was noted that they were exclusively surgical departments. This indicates that these departments conduct more blood crossmatching than necessary, primarily for preoperative cases. Overestimating the requirement of blood units preoperatively will result in underutilization of the crossmatched blood, resulting in high C/T ratios [11,12].

The utilization of the C/T ratio as a tool for analyzing the performance of the blood center has certain advantages. The C/T ratio will provide a detailed pattern or distribution of the unused crossmatched packed RBCs, categorized by department and, if necessary, tailored to specific clinicians. Wherever a high C/T ratio is observed, specific in-service training or continuing medical education (CME) programs may be implemented to address the elevated C/T ratio. Regular CMEs highlighting the best transfusion practices can ensure the proper functioning of blood transfusion services. When deciding on transfusion, the clinician must assess the necessity of the procedure and, if deemed necessary, determine the expected number of units required for the patient [3].

Given the high C/T ratio, the departments involved must be reviewed regularly. Regular auditing, preferably prospective auditing, from the blood center will be able to check such unnecessary crossmatches and even prevent transfusion of blood products that do not comply with the national transfusion guidelines. This will act as a preventive action and should be considered an important measure in the proper functioning of the blood center. Proper auditing acts as a surveillance tool with which the hospital transfusion committee can modify or alter the hospital transfusion guidelines per the requirements [13]. These evidence-based crossmatching protocols will result in significant functional improvement within the blood center.

Since the high C/T ratio was observed among the surgical departments, the introduction of MSBOS (Maximum Surgical Blood Ordering Schedule) will help prevent unnecessary crossmatches. MSBOS will identify the surgical procedures that typically would not require blood. It will also serve as a guide on the number of blood components that should be made available for each type of surgical procedure based on the departments, type of surgery, and clinician-specific blood utilization patterns. Once the MSBOS gets implemented, the blood transfusion services will be able to crossmatch the predicted number of blood units as per the planned surgical procedure. These can be altered or modified per the patient's requirements [7].

Another way to counter the high C/T ratio is by adopting the type and screen protocol policy in place of crossmatch for pre-transfusion tests. The type and screen protocol will involve the determination of the patient's ABO group and Rh type, along with screening for any unexpected and clinically significant allo-antibodies. If the antibody screen is negative, it means that the patient has no clinically significant allo-antibody, and any ABO-compatible blood from the blood center stocks can be issued to the respective patient after an immediate spin crossmatch (which is a simple test with results in approximately 10 minutes). Blood, if required, can then be issued within 10 minutes. Since blood is not being crossmatched unnecessarily and no blood unit is reserved, conducting a type and screen will serve as a safe and cost-effective alternative [14]. When the antibody screen yields a positive result, a serological crossmatch is performed to identify compatible blood units.

The limitations of this study include its short duration and the lack of analysis of clinician-dependent transfusion requirements. Additionally, the results are specific to our institution and cannot be generalized.

## Conclusions

The evaluation of the C/T ratio has shown that our hospital has yet to achieve the international standards for blood centers. The high C/T ratio was observed among certain surgical departments, implying that most of the crossmatched blood units were not being utilized effectively. Here, the C/T ratio was used as a quality indicator to analyze the utilization of blood bank resources. Optimization of the practice of blood orders is required to decrease the overordering, unnecessary blood compatibility tests, wastage of blood units due to expiry, proper management of the staff workforce, and adequate healthcare cost management. Effective patient blood management and strict adherence to transfusion guidelines are essential for the proper functioning of a blood center.
